# Developing new intermuscular coordination patterns through an electromyographic signal-guided training in the upper extremity

**DOI:** 10.1186/s12984-023-01236-2

**Published:** 2023-09-01

**Authors:** Gang Seo, Jeong-Ho Park, Hyung-Soon Park, Jinsook Roh

**Affiliations:** 1 Department of Biomedical Engineering, Cullen College of Engineering, University of Houston, Houston, TX USA; 2 Department of Mechanical Engineering, Korea Advanced Institute of Science and Technology, 291 Daehak-ro, Yuseong-gu, Daejeon, South Korea

**Keywords:** Muscle synergy, Intermuscular coordination, Myoelectric computer interface, EMG-guided exercise, Motor neurorehabilitation

## Abstract

**Background:**

Muscle synergies, computationally identified intermuscular coordination patterns, have been utilized to characterize neuromuscular control and learning in humans. However, it is unclear whether it is possible to alter the existing muscle synergies or develop new ones in an intended way through a relatively short-term motor exercise in adulthood. This study aimed to test the feasibility of expanding the repertoire of intermuscular coordination patterns through an isometric, electromyographic (EMG) signal-guided exercise in the upper extremity (UE) of neurologically intact individuals.

**Methods:**

10 participants were trained for six weeks to induce independent control of activating a pair of elbow flexor muscles that tended to be naturally co-activated in force generation. An untrained isometric force generation task was performed to assess the effect of the training on the intermuscular coordination of the trained UE. We applied a non-negative matrix factorization on the EMG signals recorded from 12 major UE muscles during the assessment to identify the muscle synergies. In addition, the performance of training tasks and the characteristics of individual muscles’ activity in both time and frequency domains were quantified as the training outcomes.

**Results:**

Typically, in two weeks of the training, participants could use newly developed muscle synergies when requested to perform new, untrained motor tasks by activating their UE muscles in the trained way. Meanwhile, their habitually expressed muscle synergies, the synergistic muscle activation groups that were used before the training, were conserved throughout the entire training period. The number of muscle synergies activated for the task performance remained the same. As the new muscle synergies were developed, the neuromotor control of the trained muscles reflected in the metrics, such as the ratio between the targeted muscles, number of matched targets, and task completion time, was improved.

**Conclusion:**

These findings suggest that our protocol can increase the repertoire of readily available muscle synergies and improve motor control by developing the activation of new muscle coordination patterns in healthy adults within a relatively short period. Furthermore, the study shows the potential of the isometric EMG-guided protocol as a neurorehabilitation tool for aiming motor deficits induced by abnormal intermuscular coordination after neurological disorders.

**Trial registration:**

This study was registered at the Clinical Research Information Service (CRiS) of the Korea National Institute of Health (KCT0005803) on 1/22/2021.

## Background

The question of how the central nervous system (CNS) coordinates the spatiotemporal activation of a group of muscles to achieve goal-directed limb movement [1] is fundamental to understanding neuromuscular control. The answer to the question can make a significant impact on motor learning, motor development, and neurorehabilitation. Previous studies have investigated that the activities of a specific group of muscles are facilitated by spinal motoneurons, whose activities are coordinated by the network consisting of the spinal premotor interneurons [2–4] and cortical motoneurons [5]. This modular organization of multi-muscle activities has also been introduced as a basis function that allows CNS to reduce the search space of motor commands [6], thereby simplifying the control of goal-directed movements [7–11].

The concept of muscle synergy has been utilized to computationally identify the recruitment of muscle groups characterizing the coordinated patterns of muscle activity which incorporate to produce various motor behaviors [12]. Adopting the dimensionality reduction methods, such as principal component analysis (PCA), independent component analysis (ICA), or nonnegative matrix factorization (NMF) [13–15], previous studies have modeled the recorded motor signals as a linear combination of time-varying (activation profile) and time-invariant (muscle synergy vector; synergy composition) components [16–21]. In human studies, it was observed that a few numbers of muscle synergies could capture the characteristics of global patterns of muscle activation underlying locomotion [22, 23], arm reaching [18, 19, 24, 25], and hand gestures [26, 27].

The concept of muscle synergies has been further applied to characterize potential changes in intermuscular coordination underlying motor development and learning [28–30]. Previous studies have established that muscle synergies can be either innate or developed early in life [28]. While a few of these preexisting muscle synergies tend to remain consistent throughout an individual’s lifespan [24], new muscle synergies are expressed during motor development in individuals of different ages, ranging from toddlers to adults [28, 30]. In addition, modification of the existing muscle synergies was observed when motor learning was achieved over a long-time span in adulthood (3–30 years) [29, 30]. Although these findings show how muscle synergies underlie motor development and learning, whether it is feasible to alter muscle synergies or develop new ones in an intended way through a relatively short-term motor exercise to improve motor output in adulthood remains unclear.

Few studies have attempted to modulate the activation profiles of muscle synergies using electromyographic (EMG) signal-guided conditioning or physical training in healthy adults [31, 32]. Torricelli et al., (2020) targeted delaying the peak of activation of one target muscle in the lower extremity by using its EMG envelope as visual biofeedback in a cycling task. Based on their results, the protocol induced a time shift in low extremity muscle synergy activation, which suggested that synergistic temporal commands were adjusted to meet the demands of short-term learning of cycling movement. Another study in the upper extremity (UE) showed improvement in the ability to modulate the activation pattern of existing muscle synergies in the hand through EMG pattern-guided training [32]. The composition of muscle synergy, however, tended to be conserved even as the control of the activation pattern was improved, which potentially implied that the nervous system may prefer utilizing habitual intermuscular coordination patterns [32–34]. Therefore, it still remains unclear whether a healthy adult can either develop new intermuscular coordination patterns (synergy vectors) or alter habitual ones in an intended way through motor training. This knowledge gap is important to investigate since targeting or normalizing muscle synergy can also benefit patients whose intermuscular coordination is disrupted following neurological injuries.

Therefore, the current study first aimed to test the feasibility of inducing the new intermuscular coordination patterns (synergy vectors) in the neurologically intact arm, which can ultimately be applied to design a stroke rehabilitation protocol. We designed an isometric EMG-guided exercise protocol to target the alteration of a habitual elbow flexor synergy in neurologically intact young adults. Over a period of six weeks, ten participants were trained to learn independent control of the activation of a pair of elbow flexor muscles that tended to be naturally co-activated. Throughout the training period, we assessed the effect of the training on the intermuscular coordination of the trained arm, the task performance, and the characteristics of individual muscles’ activity [35, 36]. Lastly, the implication of the EMG-guided isometric exercise for stroke rehabilitation was further discussed.

## Methods

### Participants

10 neurologically intact, young participants (three males; 25.3 ± 3.4 years of age) with no history of muscular or orthopedic injuries in UE participated in the study. Dominant arms were trained and assessed through six weeks of training (nine right-handed). The study was performed in accordance with the Declaration of Helsinki, with the approval of the Institutional Review Board of the University of Houston and the Korea Advanced Institute of Science and Technology. Informed consent was obtained from each participant prior to each training and assessment. The trial was registered at the Clinical Research Information Service (CRiS) of Korea National Institute of Health, KCT0005803.

### Electromyography recording

A wireless EMG recording system (Trigno Avanti Platform; Delsys Inc., Natick, MA) was used to acquire EMGs from 12 major UE muscles at a sampling rate of 1 kHz: brachioradialis (BRD), biceps brachii (medial head; BI), triceps brachii (long and lateral heads; TRIlong and TRIlat), deltoids (anterior, middle, and posterior fibers; AD, MD, and PD), pectoralis major (clavicular fibers; PECT), trapezius (upper, middle, and lower fibers; UpTrp, MidTrp, and LowTrp), and infraspinatus (InfSp). Through the system, EMGs were bandpass filtered (20–450 Hz) and amplified (x1000). The wireless EMG sensors were placed on each muscle’s belly in accordance with the guidelines provided in the Surface Electromyography for the Non-Invasive Assessment of Muscles (SENIAM)–European Community project [37, 38]. To keep the consistency of the sensor placement throughout six weeks of training, a long sleeve compression shirt was customized for each participant to have an opening at the location of each muscle’s belly.

**Fig. 1:**
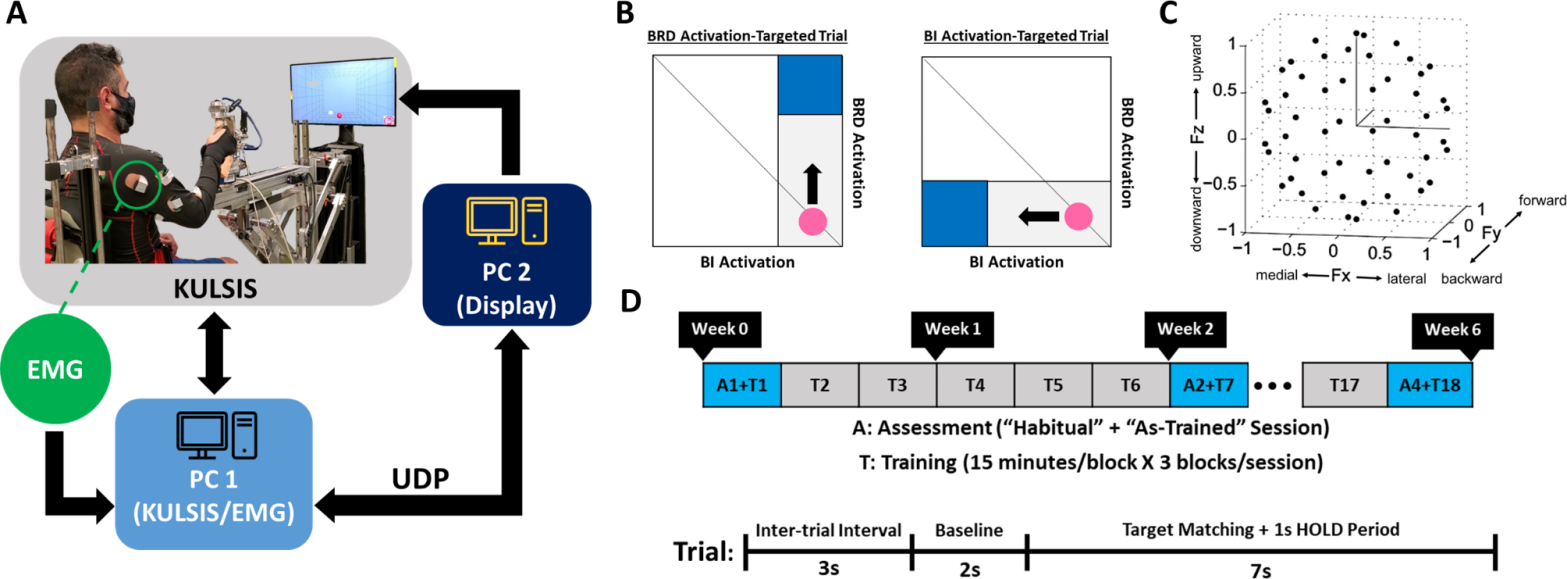
The overview of the experimental setup and study design. **A**, KAIST Upper Limb Synergy Investigation System (KULSIS). The isometric force and the electromyography (EMG) signal were acquired and processed by the main computer (PC1). The information for the display (force data for the assessment and EMG data for the training) was transmitted from PC1 to the display computer (PC2) via User Datagram Protocol (UDP). **B**, The EMG-guided training paradigm. The activation amplitudes of the targeted muscle pair, the brachioradialis (BRD) and biceps brachii (BI), were mapped to the vertical and horizontal displacement of a cursor, respectively. The cursor remained at the right bottom corner of the display when there was no activation of both targeted muscles. Participants were verbally guided to generate an appropriate force to move the cursor within the elongated squared region to reach the blue target zone and remain in the zone for 1 s consecutively to make a successful target match. The area of the square target zone was defined as 30% of the target activation level of each muscle (70% of the maximum voluntary contraction (MVC)). The diagonal line indicates the condition when both targeted muscles are activated together to an equal extent. **C**, The spatial distribution of 54 normalized force targets for the assessment of the proposed exercise effects. Fx, Fy, and Fz directions represent medial-lateral, backward-forward, and downward-upward directions, respectively. **D**, Timeline of the six-week training and assessment. The study consisted of 18 sessions of training (three sessions per week) and four assessments (at Weeks 0, 2, 4, and 6). Each assessment included both “Habitual” and “As-Trained” conditions. For each trial of both training and assessment, 3 s of an inter-trial interval, 2 s of a baseline period, and up to 7 s of a target matching window were provided for task completion

### Isometric training and assessment setup

For the isometric training and assessment, a custom-designed device, KAIST Upper Limb Synergy Investigation System (KULSIS) [39] with a six-degree-of-freedom load cell was used (Fig. 1A). The three-dimensional end-point forces generated at a gimbal handle mounted on the load cell were recorded at a sampling rate of 1 kHz. The analog signals of force and EMG were recorded simultaneously and digitized before being synchronized using an internal clock and the markers, controlled by customized software written in LabVIEW (National Instruments, Austin, TX). The main computer used to collect the forces and the EMG signals and run the main LabVIEW software was connected to a display computer via User Datagram Protocol (UDP) to visualize targets and a cursor on a screen for the participants through Unity 3D (Unity Technologies, San Francisco, CA) software for the assessment and a LabVIEW software for training (Fig. 1A).

The vertical and horizontal location of the center point of the KULSIS handle was aligned with the acromion of the sitting participant’s dominant UE. The distance between the center of the handle and the shoulder was adjusted to 60% of the arm’s full length. The angles of elbow flexion (acromion-lateral epicondyle-the center of the palm; 80°-90°), shoulder flexion (axis parallel to the trunk-acromion-lateral epicondyle; 30°-40°), and shoulder abduction (axis parallel to the spinal cord-acromion-olecranon; 10°-20°) were also measured using a digital goniometer prior to each experiment in order to keep the consistency of the posture across different training and assessment sessions. After the adjustment of UE posture, the trunk posture and upper body movement of the participant were constrained using a harness attached to the KULSIS seat. During the task, the operator closely monitored the participants’ motor performance and provided verbal instructions, if needed, to maintain a consistent arm posture throughout the training and assessment sessions. Any trials that included noticeable movement of the upper extremities were excluded and substituted with additional make-up trials.

### Isometric EMG-guided training

The six-week isometric EMG-guided training consisted of 18 sessions (three sessions per week, spaced every other day). In each session, the participant was trained to control a cursor, whose horizontal and vertical movement in a two-dimensional square space was mapped to the activation magnitude of EMGs recorded from BRD and BI, respectively, while holding the KULSIS handle in a sitting posture as described in the previous section. The major objective of the training was to develop motor strategies to intentionally activate the activation-targeted (AT) muscle while restraining the suppression-targeted (ST) muscle, where AT and ST muscles were randomly selected for each trial. For a BRD-AT trial, BRD and BI were AT and ST muscles, respectively, and vice versa for a BI-AT trial. In order to successfully match a target, the participant needed to strategically modulate muscle activation to drive the cursor from starting position (right bottom corner) to a square target zone, which appeared in either the upper right corner (BRD target) or lower left corner (BI target) of the square space, and hold the cursor in the zone for 1s (HOLD period) (Fig. 1B). The size of the square space was determined based on the EMG amplitude of each targeted muscle measured during maximum voluntary contraction (MVC) prior to each training session. The horizontal and vertical scales of the square were normalized with 70% MVC level of BI and BRD, respectively. The dimension of the target zone (the blue zone in Fig. 1B) was defined as 30% of the square space. For each training trial, up to 7 s of target matching time including 1 s of the HOLD period were given after 2 s of the baseline period. In the case when the participant failed to maintain the cursor in the target zone for 1 s within the given target matching time, the trial was marked as an unmatched trial. Each session was an hour long and consisted of three blocks of 15-minute training and a 5-minute break in between. The participants were instructed to match the targets as many as possible in each block (Fig. 1D).

### Isometric force matching assessment

To assess changes in the intermuscular coordination pattern, the participants performed an isometric reaching, untrained during 18 training sessions, in a three-dimensional virtual force space using KULSIS at Week 0 (pre-training), 2, 4, and 6 (post-training) (Fig. 1C and D). Sitting on the KULSIS seat with the same posture as instructed for the training, the participants generated the forces on the handle of the device to drive a cursor toward one of 54 different target directions on the screen (Fig. 1C). The force targets were uniformly distributed on the surface of a unit sphere to prevent any bias in force generation. In each assessment trial, a target was given in random order. The participant controlled the force-driven cursor to the target and held it within a logical radius (20% of the targeted force magnitude) of the target for 1 s (HOLD period). After 2 s of the baseline period, the participant had up to 7 s for a target match including the HOLD period to complete the trial. The targeted force magnitude for each target (the radius of the unit sphere) was personalized as 40% of the maximum lateral force (MLF). MLF, which was empirically determined as the weakest maximum force direction in space for most of the participants, was measured under the same isometric condition using KULSIS before each assessment.

The isometric force matching assessment consisted of two conditions: (1) “Habitual” and (2) “As-Trained” conditions. In the “Habitual” condition, the participants were instructed to utilize their natural motor strategies, as they did prior to the isometric exercise, to match the target in the force space. For the “As-Trained” condition, the verbal instruction was to consciously apply the trained motor strategies acquired to isolate BRD and BI activation from each other during the isometric exercise. For the task performance at Week 0 under the “As-Trained” condition, general verbal guidance determined empirically was provided for the isometric exercise because no new, specific motor strategies were not developed yet. (The verbal guidance for facilitating BI activation was “squeeze the handle in an outward direction during force generation in medial directions”. For BRD activation, verbal guidance, “squeeze the handle in an inward direction during force generation in upward directions”, was given prior to the assessment.) The participants who could activate BRD and BI in isolation with the verbal guidance prior to any training sessions were excluded from the study enrollment.

### Signal-to-signal ratio (SSR) analysis

To quantify the training effect on the activation of targeted muscle pair, BRD and BI, signal-to-signal ratio (SSR), a ratio between the root-mean-squared (RMS) EMG amplitude of two muscles, was calculated for each training session as follows:

1 $$SSR={\left(\frac{RMS \left(\left|{EMG}_{AT}\right|/\text{m}\text{a}\text{x}\left(\right|{EMG}_{AT}\left|\right)\right)}{RMS \left(\left|{EMG}_{ST}\right|/\text{m}\text{a}\text{x}\left(\right|{EMG}_{ST}\left|\right)\right)}\right)}^{2}$$

where EMG_AT_ was concatenated HOLD period EMGs of the AT muscle across matched trials, while EMG_ST_ was of the ST muscle. SSR was calculated separately for BRD-AT trials and BI-AT trials. All the HOLD period EMGs were demeaned and rectified prior to being concatenated. The concatenated EMG amplitude per muscle was normalized by its maximum value.

### Resting period EMG frequency analysis

In order to explore the changes in the frequency-domain feature of the EMGs after training, the mean frequency of each UE muscle’s EMG, recorded during the baseline period of the training trials, was identified from the first two weeks and the last two weeks of the training. The EMGs collected from the baseline period of each trial were demeaned and concatenated across all the trials for each training session. For the frequency analysis, both matched and unmatched trials were included because of no differences in their baseline EMG attributes and more data points for frequency-domain analysis. The power spectrum of the concatenated EMG of each muscle was analyzed using a fast Fourier transform algorithm, and the normalized mean frequency was calculated for each muscle [40, 41].

### Muscle synergy identification

Muscle synergies were identified from the EMGs recorded during isometric force matching assessment using a non-negative matrix factorization (NMF) method [16, 42, 43]. Prior to the synergy identification, the raw EMG data of each trial were filtered using the level-7 sym4 wavelet decomposition and reconstruction method to suppress the electrocardiogram (ECG) artifacts. Following the ECG denoising, the EMGs were demeaned to remove the DC offset, and the envelope of the processed EMG signal was obtained through full-wave rectification and low-pass filtering (4th order Butterworth filter with cutoff frequency at 10 Hz). To obtain task-relevant activation of each muscle, a force onset, where the magnitude of the forces exceeded the three standard deviations of the mean baseline force magnitude, was identified for each trial. Also, both force and EMG data were segmented from the onset point to the end. After the segmentation, the EMGs of the 54 trials in any assessment session (see Sect. 2.3) were concatenated as a single matrix that reflected the characteristics of intermuscular coordination during the three-dimensional isometric reaching. Lastly, the concatenated EMGs were normalized to have a unit variance to minimize any potential bias in synergy identification toward high-variance EMGs [43].

Using the NMF algorithm, the pre-processed EMGs were reconstructed as a linear combination of a muscle synergy set (*W*) and its corresponding activation coefficients (*C*) [16, 17, 44–46],

2 $$EM{G_{reconstructed}}\, = \,W\, \cdot \,C$$

, where *W* was an *M* (the number of muscles) by *S* (the number of muscle synergies) matrix, and *C* was an *S* by *N* (the number of data samples) matrix. For a given S, W and its corresponding *C* were identified from a randomly selected 60% of the given EMG data, and the remaining 40% was reconstructed using the selected subset [43, 46]. After 100 repetitions of the identification-reconstruction process, the muscle synergy set with the highest global variance-accounted-for (gVAF) value was selected among the 100 sets for further analysis. The VAF value was defined based on the ratio between the summation of the squared errors (SSE) and the total sum of the squares (SST) of the EMGs, which can be expressed as

3 $$VAF\left(\%\right)=100\times \left(1-\frac{SSE}{SST}\right)$$.

To estimate the optimal *S* given an EMG dataset, a gVAF value and a difference in gVAF (diffVAF), which was acquired by adding an additional synergy to a given *S*, were utilized as criteria. The *S* that satisfied gVAF > 90% and diffVAF < 3% was defined as the estimated optimal number of muscle synergies. The change in the composition of muscle synergy was quantified using a similarity score calculated through a scalar product of *W*s in comparison [43, 47]. The mean score of the entire muscle synergy set, as well as the mean score of the trained muscles-dominant (BRD & BI) synergies, were analyzed across the weeks for each of the two assessment conditions (i.e., “Habitual” and “As-Trained”). For the *C*, the mean value of each trial was calculated to obtain an activation profile of their corresponding *W*. Furthermore, each of *C* was multiplied by the normalized (by a normalization factor, the force vector magnitude) force components to examine the force mapping of the activation profile.

### Training outcome measurements

In addition to the attributes of intermuscular coordination, the effects of the isometric exercise on the motor control of the targeted muscles were assessed at each training session by calculating the following metrics: the number of targets matched, target matching time (task completion time after the baseline period), the total path length of the cursor, the average velocity of the cursor, and the root-mean-square error (RMSE) of the coordinates of the cursor with respect to the target coordinates. The analysis included the matched trials only, and the trials were analyzed separately based on each of the targeted muscles (BRD and BI). The total path length and RMSE were defined as a summation of the displacement of consecutive cursor positions, (*P*_*x*_(n), *P*_*y*_(n)), and a summation of the distance between the cursor position at the n^th^ sampling and the coordinates of the target ((*P*_Tx_, *P*_Ty_); BRD: (0,1), BI: (1,0)) divided by the total number of data points (*N*), respectively:

4 $$Total\,Path\,Length\, = \,\sum\nolimits_{n = 2}^N {\sqrt {{{\left( {{P_x}\left( n \right) - {P_x}\left( {n - 1} \right)} \right)}^2} + {{\left( {{P_y}\left( n \right) - {P_y}\left( {n - 1} \right)} \right)}^2}} }$$

5 $$RMSE =\sqrt{\frac{\sum _{n=1}^{N}\left({({P}_{x}\left(n\right)-{P}_{Tx})}^{2}+{({P}_{y}\left(n\right)-{P}_{Ty})}^{2}\right)}{N}}$$

#### Statistical analyses

To test the normality of data distribution, the Kolmogorov-Smirnov test (α = 0.05) was used, and the estimated number of muscle synergies satisfied the condition of normal distribution while the rest of the outcome measurements had non-normal distribution. Therefore, a parametric test, such as two-way ANOVA, was used to test the statistical significance of the change in the estimated number of muscle synergies across the assessment conditions and weeks. The non-parametric test, the Wilcoxon Rank-Sum test with Bonferroni correction, was used to test the rest except for the similarity score of the muscle synergies.

The significance of the similarity score of the muscle synergies in comparison was tested by computing the similarity score of all possible pairs of a random synergy set, 1000 synergies randomly selected from the entire synergy sets identified in this study. The 95th percentile level of the similarity scores computed from the random synergy sets was used as a similarity threshold (Th_sim_ = 0.78) [43, 46, 48, 49] to determine the significance of a similarity score of muscle synergies.

## Results

### Improvement of neuromotor control of the trained muscles

**Fig. 2:**
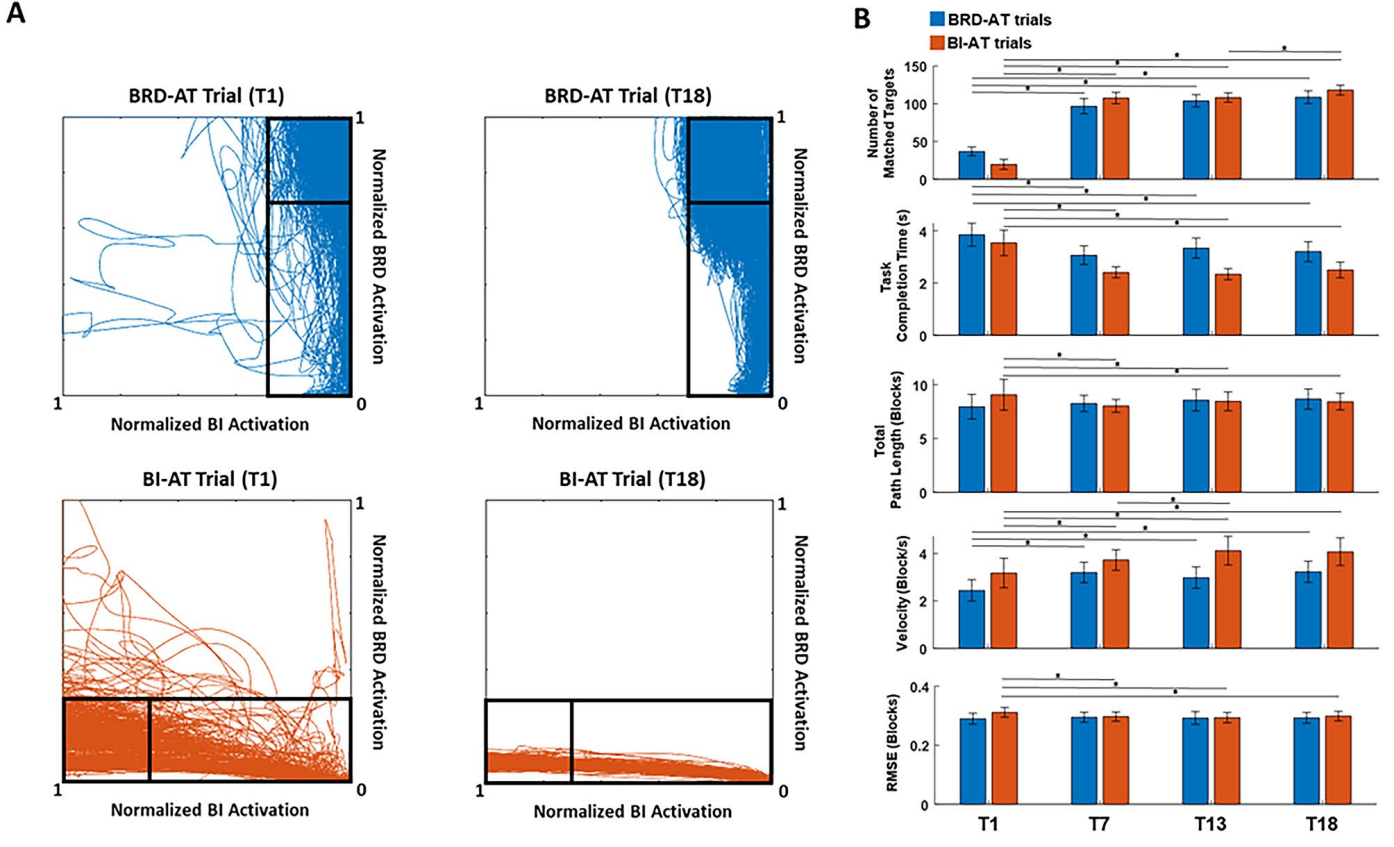
Changes in motor task performance after the isometric exercise. **A**, Cursor trajectories reflecting EMG measured during a representative session of a participant in the first (T1) and the last (T18) session of the training. **B**, The number of matched targets, task completion time, total path length, velocity, and RMSE of cursor movement per training session in brachioradialis (BRD) activation-targeted (AT) and biceps (BI)-AT trials in blue and red, respectively (mean ± standard error; Wilcoxon Rank-Sum test; *, *p* < 0.05)

During the six-week isometric training, improvements in the neuromotor control of the trained muscles were observed (Fig. 2). As shown in the representative figure of the cursor trajectory tracked during the first and the last training session, the participants showed noticeable progress in driving the cursor into the target zone by modulating the activation of targeted muscle more efficiently (Fig. 2A). At the 7th session of the training (T7; after completion of Week 2), the velocity of the cursor movement increased (T1: 3.2 ± 0.63 blocks/s; T7: 3.7 ± 0.43 blocks/s; mean ± standard error (SE), *p* = 0.045) while the total path length decreased (T1: 9.1 ± 1.4 blocks; T7: 8.1 ± 0.61 blocks; mean ± SE, *p* = 0.047), which resulted in a significant reduction of task completion time (T1: 3.5 ± 0.49 s; T7: 2.4 ± 0.21 s; mean ± SE, *p* = 0.034) and an increase in the number of targets matched (T1: 19 ± 6.8 targets; T7: 110±7.6 targets; mean ± SE, *p* < 0.01) for BI-activation-targeted (BI-AT) trials (Fig. 2B). In addition, there was a significant improvement in RMSE of the cursor movement at T7 for BI-AT trials (T1: 0.31 ± 0.016 blocks; T7: 0.29 ± 0.010 blocks; mean ± SE, *p* < 0.41). A similar trend of changes in the outcome measures of training was observed in BRD-AT trials, while decreases in the total path length (T1: 7.9 ± 1.1 blocks; T7: 8.3 ± 0.8 blocks; mean ± SE) and RMSE (T1: 0.28 ± 0.010 blocks; T7: 0.30 ± 0.011 blocks; mean ± SE) were not observed (Fig. 2B). After T7, the level of task performance during BRD-AT trials was maintained, whereas a continuous improvement of task performance (number of targets matched and velocity) during BI-AT trials was achieved for the rest of the training period.

**Fig. 3:**
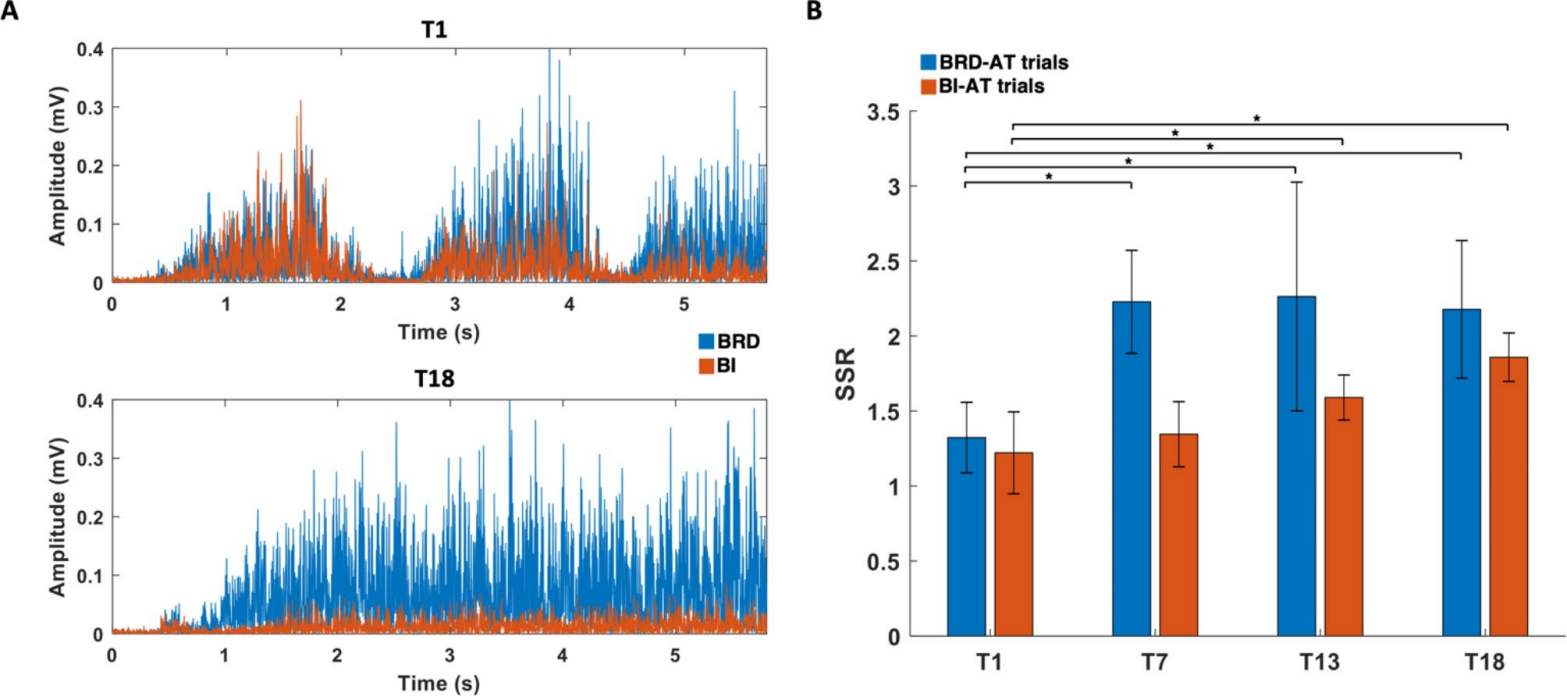
Raw EMGs of the targeted muscles and their signal-to-signal ratio (SSR) across the training sessions. **A**, Rectified EMG signals of brachioradialis (BRD; blue) and biceps brachii (medial head, BI; red) collected during trials of the first (T1) and last (T18) training sessions from a representative participant’s data. **B**, SSR between the activation-targeted (AT) and suppression-targeted (ST) muscle measured at T1 (first training), T7 (after two weeks of training), T13 (after four weeks of training), and T18 (last training) for each of BRD-AT and BI-AT trials (Wilcoxon Rank-Sum test; *, *p* < 0.05)

Through the six weeks of training, the participants could intentionally activate an AT muscle while suppressing the activation of the ST muscle under the isometric condition. As shown in the representative, raw EMG data collected from a participant during the first and the last session of training (Fig. 3A), the ST muscle (BI), which was persistently co-activated with the AT muscle (BRD) at the first training session, showed activation noticeably decoupled from the AT muscle after the training. Reflecting this learning of activation decoupling through the training, the SSR analysis quantified a significant increase in the ratio of the normalized amplitudes of the AT muscle to the ST muscle (Fig. 3B). Specifically, the mean SSR of BRD-AT trials increased significantly after two weeks of training (T1: 1.3 ± 0.22; T7: 2.3 ± 0.31; mean ± SE, *p* = 0.026) and maintained the level for the rest of the training period, while the increase in mean SSR of BI-AT trials was not statistically significant until the moment after four weeks of training (T1: 1.2 ± 0.31; T13: 1.6 ± 0.12; mean ± SE, *p* = 0.046). Although the participants required more training to isolate BI activation from BRD activation, a continuous increase in the mean SSR of BI-AT trials was observed throughout the six weeks of training.

### Consistency of the number of muscle synergies across the training weeks and assessment conditions

**Fig. 4:**
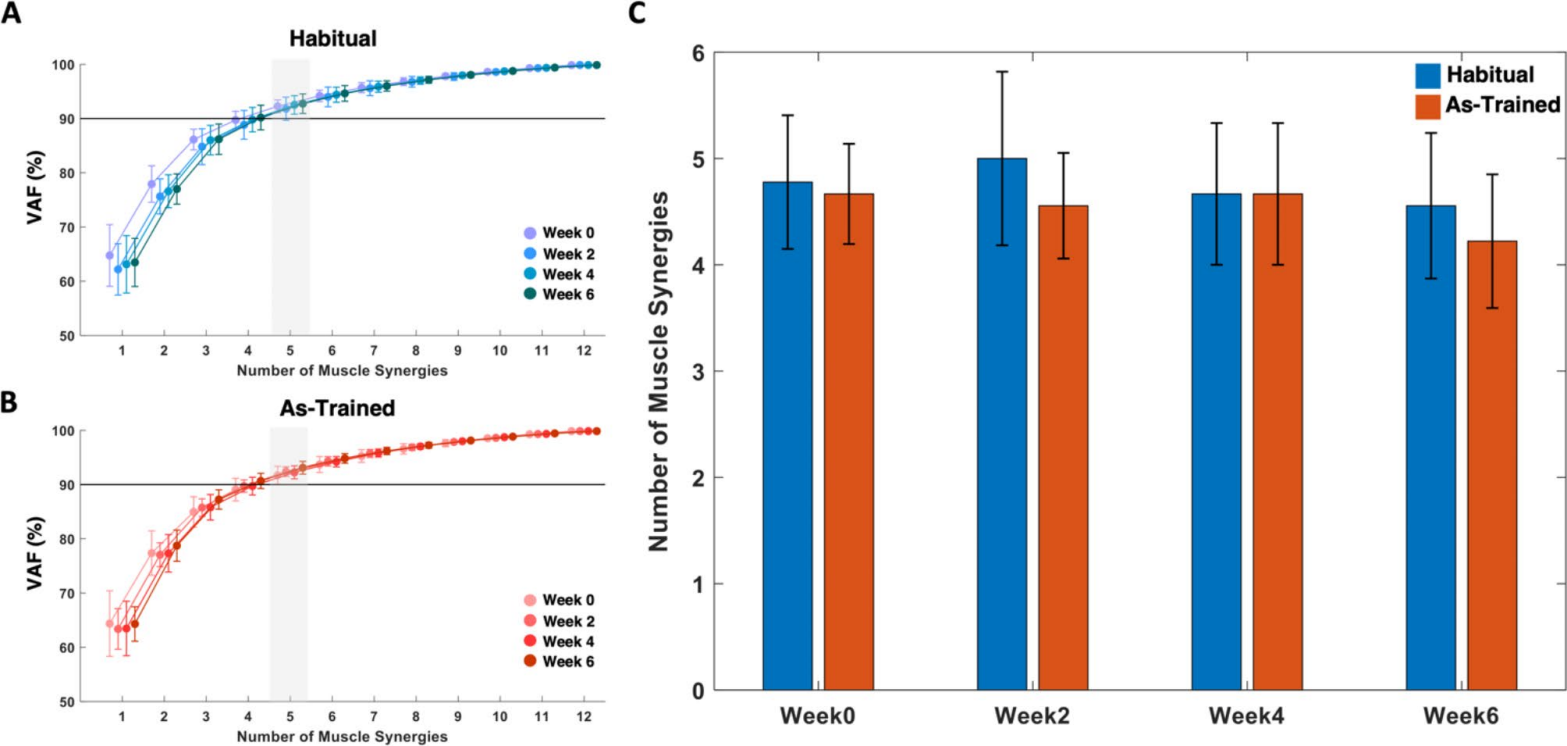
The number of muscle synergies estimated based on variance-accounted-for (VAF). **A-B**, The mean and SD of VAF across the participants at each assessment week for the “Habitual” (**A**, blue) and “As-Trained” conditions (**B**, red). The number of muscle synergies where the group mean VAF exceeds the VAF threshold at 90% (black line) is highlighted (grey). **C**, The mean and SD of the number of muscle synergies that can explain at least 90% of the total variance of raw EMGs across the participants

When the muscle synergies were identified from the 12 muscles at each assessment under “Habitual” and “As-Trained” conditions, five synergies were typically required throughout the training weeks across the participants (Fig. 4). This optimal number of muscle synergies estimated based on the VAF level was consistent across the conditions. Compared to when the participants used their habitual motor strategies, they tended to express a smaller number of synergies when they were instructed to utilize the trained strategies, though the difference in the synergy number was statistically insignificant (As-Trained, Week0: 4.7 ± 0.47; Week2: 4.6 ± 0.49; Week4: 4.7 ± 0.67; Week6: 4.2 ± 0.63; Habitual, Week0: 4.8 ± 0.63; Week2: 5.0 ± 0.82; Week4: 4.7 ± 0.67; Week6: 4.6 ± 0.68; α = 0.05).

### Changes in the composition of muscle synergies interim and after six weeks of training

**Fig. 5:**
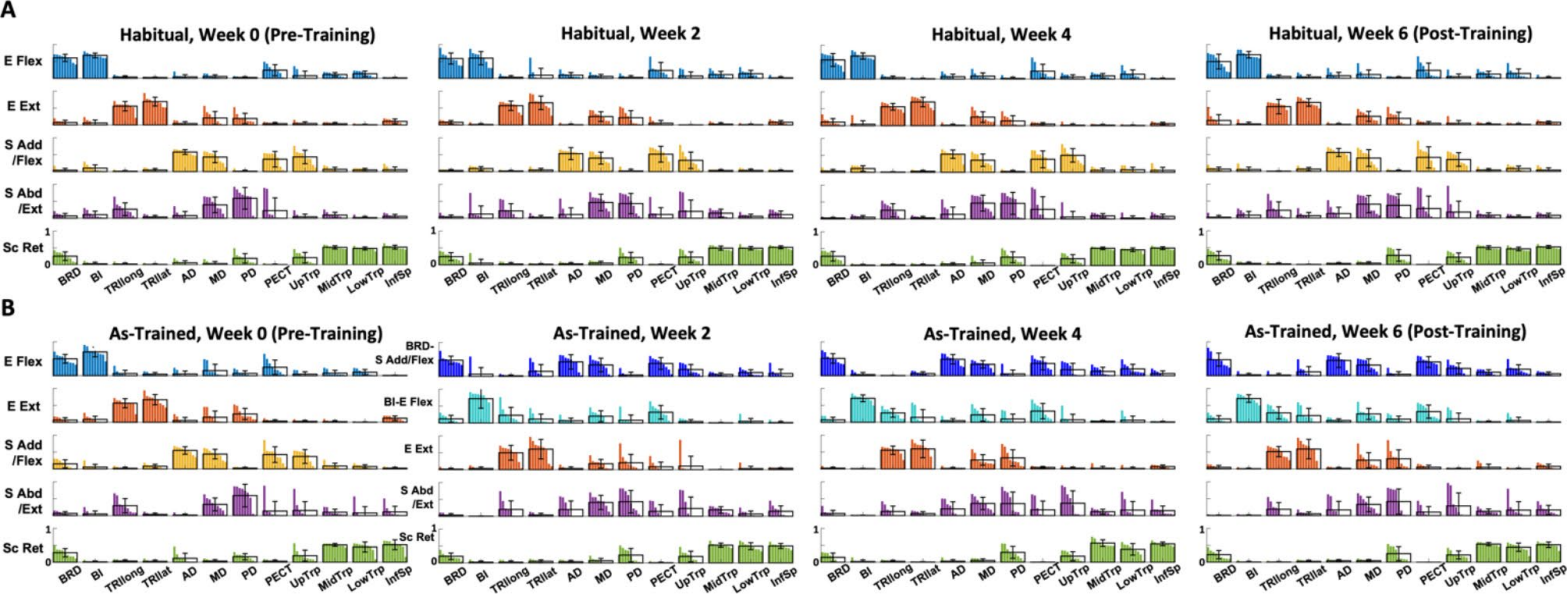
Changes in the composition of muscle synergies during six weeks of training. The mean and SD of muscle weights, superimposed on the distribution of the muscle weights of participants (n = 10), per each of five synergies identified in the assessment session at Weeks 0, 2, 4, and 6. For each muscle, the weight distribution was displayed in descending order. The recorded 12 muscles included brachioradialis (BRD), biceps brachii (medial head; BI), triceps brachii (long and lateral heads; TRIlong and TRIlat), deltoids (anterior, middle, and posterior fibers; AD, MD, and PD), pectoralis major (clavicular fibers; PECT), trapezius (upper, middle, and lower fibers; UpTrp, MidTrp, and LowTrp), and infraspinatus (InfSp). **A**, Five muscle synergies, identified when forces were generated isometrically and habitually (not using practiced motor skills; “Habitual” condition), included (1) elbow flexor (E Flex; BRD, BI, and PECT), (2) elbow extensor (E Ext; TRIlong and TRIlat), (3) shoulder adductor/flexor (S Add/Flex; AD, MD, PECT, and UpTrp), (4) shoulder abductor/extensor (S Abd/Ext; MD, PD, and TRIlong), and (5) scapula retractor (Sc Ret; MidTrp, LowTrp, and InfSp). **B**, Five muscle synergies, identified when forces were generated isometrically using practiced motor skills (“As-Trained” condition). A newly acquired muscle synergy set emerged from Week 2 included (1) a combination of BRD and S Add/Flex (BRD-S Add/Flex), (2) BI dominant E Flex with minor PECT (BI-E Flex), (3) E Ext, (4) S Abd/Ext, and (5) Sc Ret

While the number of muscle synergies was consistent across the weeks and the two assessment conditions, the structure of the synergy composition was modulated as the participants developed their own strategies to isolate the activation of the targeted muscles from each other (Fig. 5). Before the training (Fig. 5A), the five muscle synergies identified under the “Habitual” and “As-Trained” conditions shared common compositions: (1) Elbow Flexor (E Flex; BRD, BI, and PECT), (2) Elbow Extensor (E Ext; TRIlong and TRIlat), (3) Shoulder Adductor/Flexor (S Add/Flex; AD, MD, PECT, and UpTrp), (4) Shoulder Abductor/Extensor (S Abd/Ext; MD, PD, and TRIlong), and (5) Scapula Retractor (Sc Ret; MidTrp, LowTrp, and InfSp). However, as the participants started developing the motor strategies to decouple the activation of the targeted muscle pair from Week 2, the significant changes in the composition of muscle synergies, particularly the targeted muscle-related synergies, were manifested during the “As-Trained” assessment session (Fig. 5B). The newly acquired muscle synergy set included (1) a combination of BRD and S Add/Flex (BRD-S Add/Flex), (2) BI dominant E Flex with minor PECT (BI-E Flex), (3) E Ext, (4) S Abd/Ext, and (5) Sc Ret. Interestingly, BRD was decoupled from BI and formed a new synergy, as being merged with S Add/Flex. Meanwhile, BI with PECT formed a new E Flex synergy. The rest of the synergies, E Ext, S Abd/Ext, and Sc Ret, remained typically consistent. Once the new patterns of intermuscular coordination were established, the synergy set was retained for the rest of the six weeks of the training. Despite the alterations in the trained muscle synergies, the composition of “habitual” muscle synergies (i.e., the synergies activated habitually to perform required motor tasks before developing new synergies) was conserved throughout the entire training period.

**Fig. 6:**
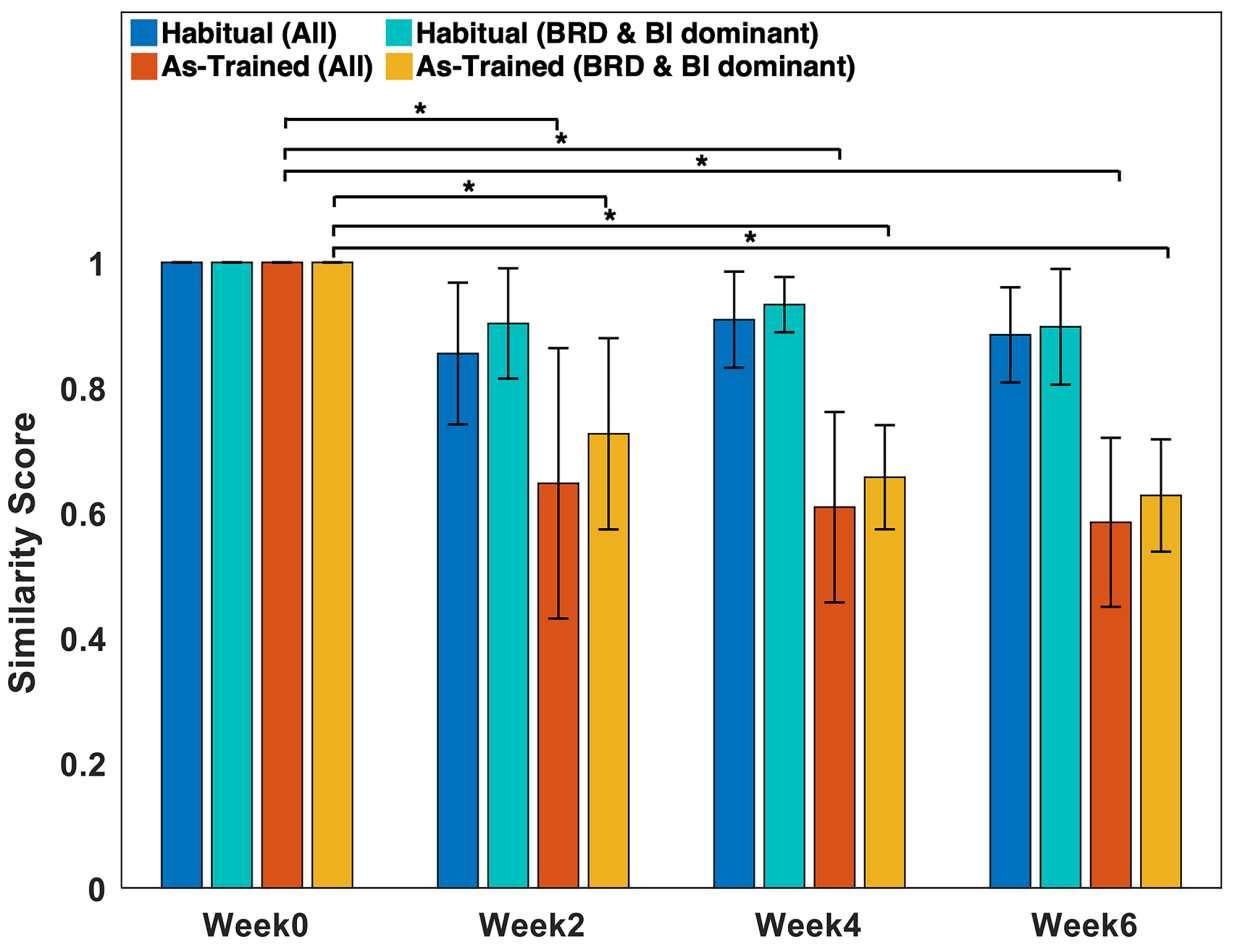
The similarity of muscle synergy composition between Week 0 and Weeks 2, 4, and 6. Blue and red represent when all the synergies were considered in “Habitual” and “As-Trained” assessment conditions, respectively. Also, cyan and yellow represent when only the BRD- & BI-dominant synergies were considered in “Habitual” and “As-Trained” assessment conditions, respectively (mean $\pm$ SD; Similarity threshold (0.78); *, *p* < 0.05)

Based on the result of the similarity analysis shown in Fig. 6, the general composition of muscle synergies expressed during the “As-Trained” session after Week 2 was significantly different from the pre-training synergy composition (Week 2: 0.65 ± 0.22; Week 4: 0.61 ± 0.15; Week 6: 0.59 ± 0.14; Th_sim_ = 0.78, *p* < 0.05). The dissimilarity of the composition was conserved even when only the targeted muscle-dominant synergies were considered for the analysis (Week 2: 0.73 ± 0.15; Week 4: 0.66 ± 0.083; Week 6: 0.63 ± 0.090; Th_sim_ = 0.78, *p* < 0.05). Once the new pattern of intermuscular coordination was developed, the pattern remained consistent for the rest of the training period.

### Characteristics of the muscle synergy activation profiles and their directional tuning

**Fig. 7:**
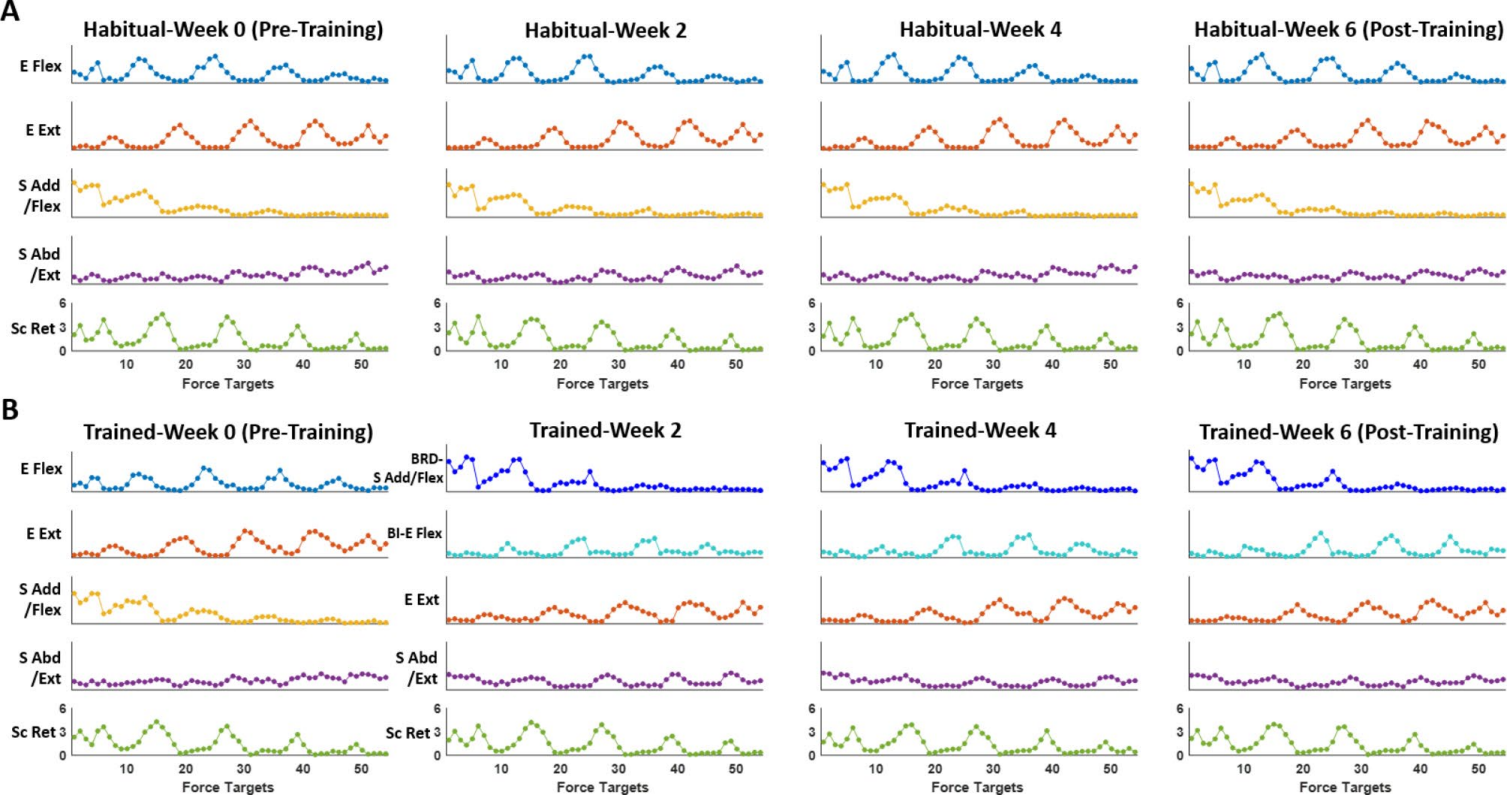
Changes in the activation profile of muscle synergies during six weeks of training. **A**, Group mean (n = 10) activation coefficients of five synergies calculated for each target of the “Habitual” condition of the assessment. Five muscles synergies that composed the habitual muscle synergy set (see Fig. 5A) included (1) elbow flexor (E Flex; BRD, BI, and PECT), (2) elbow extensor (E Ext; TRIlong and TRIlat), (3) shoulder adductor/flexor (S Add/Flex; AD, MD, PECT, and UpTrp), (4) shoulder abductor/extensor (S Abd/Ext; MD, PD, and TRIlong), and (5) scapula retractor (Sc Ret; MidTrp, LowTrp, and InfSp). **B**, Group mean (n = 10) activation coefficients of muscle synergy set obtained during the “As-Trained” condition of the assessment which included new synergy sets from Week 2: (1) combination of BRD and S Add/Flex (BRD-S Add/Flex), (2) BI dominant E Flex with minor PECT (BI-E Flex), (3) E Ext, (4) S Abd/Ext, and (5) Sc Ret (see Fig. 5B).

**Fig. 8:**
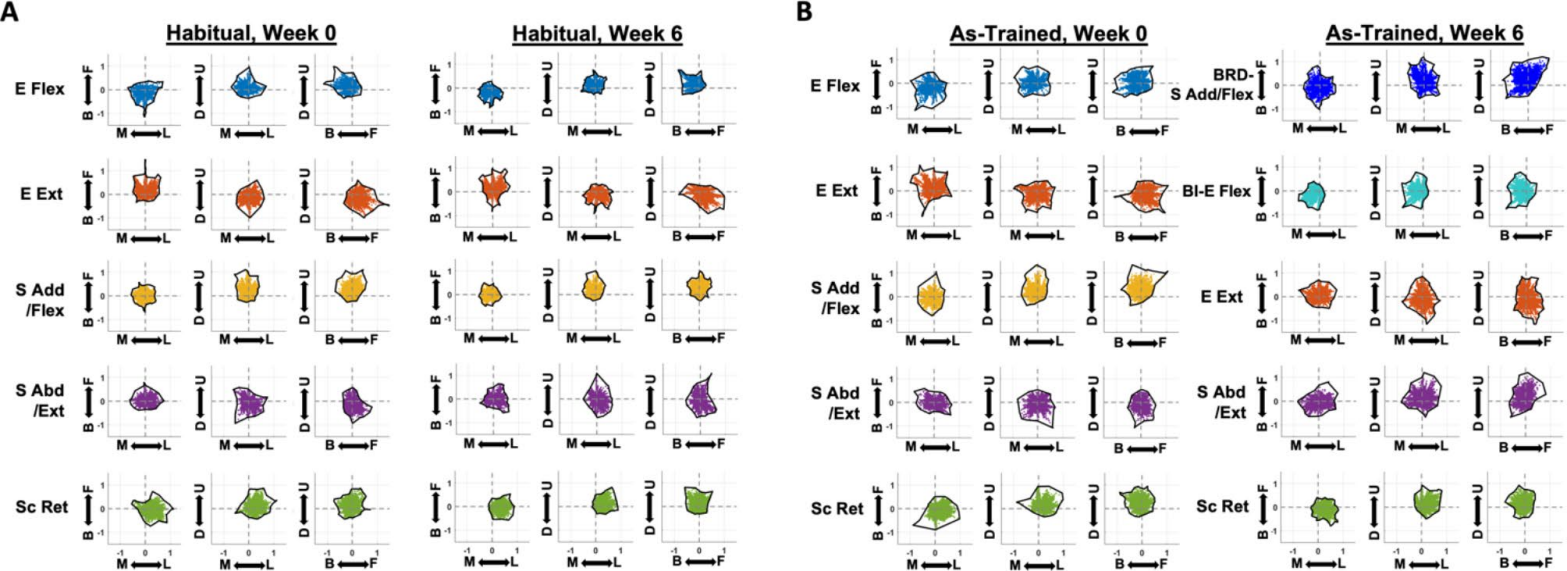
The tuning curves of the synergy activation profiles as a function of end-point forces. The tuning curves are projected on the horizontal (medial(M)-lateral(L) & forward(F)-backward(B)), frontal (M-L & upward(U)-downward(D)), and sagittal plane (F-B & U-D). The set of five synergies in the “Habitual” condition in pre- and post-training (Week 0 and Week 6, respectively) and in the “As-Trained” condition in pre-training included: elbow flexor (E Flex; BRD, BI, and PECT), elbow extensor (E Ext; TRIlong and TRIlat), shoulder adductor/flexor (S Add/Flex; AD, MD, PECT, and UpTrp), shoulder abductor/extensor (S Abd/Ext; MD, PD, and TRIlong), and scapula retractor (Sc Ret; MidTrp, LowTrp, and InfSp). The set of five synergies activated in the “As-Trained” condition in post-training included: a combination of BRD and S Add/Flex (BRD-S Add/Flex), BI dominant E Flex with minor PECT (BI-E Flex), E Ext, S Abd/Ext, and Sc Ret. **A** and **B**, The tuning curves in “Habitual” and “As-Trained” assessment conditions, respectively

The activation profile (Fig. 7) and its directional tuning (Fig. 8) reflected the biomechanical actions of the muscles activated within each synergy vector in both “Habitual” and “As-Trained” conditions. Habitually, E Flex and E Ext were activated antagonistically during backward-medial-upward reaching and forward-downward isometric reaching, respectively, while S Add/Flex and S Abd/Ext mainly contributed to upward and downward isometric reaching, respectively. Sc Ret, in general, was utilized for lateral-upward force generation (Figs. 7 and 8 A). These activation profiles of habitual muscle synergies were conserved throughout the six weeks of training (Fig. 7A). As BRD was activated independently of the BI activation and formed a new synergy with S Add/Flex synergy at Week 2, the new synergy, BRD-S Add/Flex, and the original S Add/Flex shared a similar trend of activation profile and the directional tuning (Figs. 7B and 8B). The activation profile of the other newly formed synergy, BI-E Flex, mimicked the features of the activation profile of the naturally existing E Flex, but was more tuned to generating isometric force in the medial direction (Fig. 8B). Once the new pattern of synergy activation was established, the newly formed activation profile remained consistent for the rest of the six-week training period (Fig. 7B).

### Increase in mean frequency of baseline-period raw EMGs of muscles involved in the newly established synergy

**Fig. 9:**
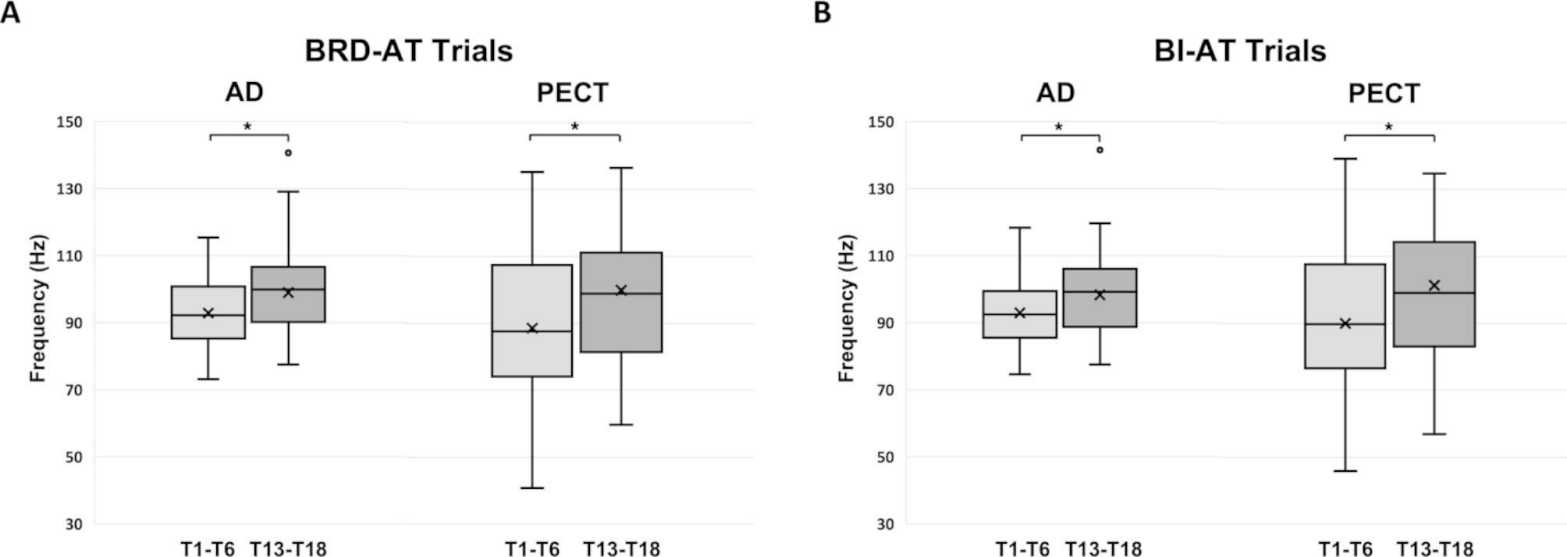
Changes in the mean frequency of recorded baseline EMGs during training sessions. The group average of the first six sessions (T1-T6) and the last six sessions (T13-18) of the training were compared. **A** and **B**, The mean frequencies of baseline EMGs recorded from the anterior deltoid (AD) and pectoralis major (PECT) during the baseline period of brachioradialis-activation-targeted (BRD-AT) trials and biceps brachii (BI)-AT trials, respectively (Wilcoxon Rank-Sum test; *, *p* < 0.05)

Not only the temporal characteristics of the muscle activation during the task performance, but also the attributes of raw EMG activation during a baseline period were also altered in the frequency domain after the training. Figure 9 shows that the mean frequency of baseline-period raw EMGs of AD and PECT, the two muscles involved in forming the newly developed synergy, increased significantly (*, *p* < 0.05) in comparison between the early phase (T1-T6) and the later phase (T13-T18) of the training. This mean frequency shift was observed only in AD and PECT during the baseline of both BRD- and BI-AT trials.

## Discussion

The current work is the first to demonstrate the feasibility of expanding the repertoire of intermuscular coordination patterns by developing new intermuscular coordination through an isometric EMG-guided exercise in neurologically intact individuals. The EMG-guided training aimed to activate the two major muscles in the elbow flexor synergy, BI and BRD, in isolation from each other to develop new muscle co-activation patterns. As a result, typically from Week 2 out of the six weeks of the training, young, healthy participants could use newly learned muscle synergies during the assessment when they were requested to perform new, untrained motor tasks by activating their muscles in the trained way (“As-trained” condition). The newly acquired muscle synergy set included a combination of BRD and the habitual S Add/Flex and a BI-dominant E Flex synergy, which could be interpreted as the dissociation of BRD from the habitual E Flex and annexation of BRD to the habitual S Add/Flex based on the results of the analysis on the synergy activation profiles. Once the participants developed new synergistic muscle activations, the newly acquired patterns were retained for the rest of the training period. Meanwhile, habitual muscle synergies, the synergistic muscle activation groups that the same participants used before the training, were conserved throughout the entire training period. The alteration in the targeted muscle synergy was also reflected in the increased ratio between the activation of activation-targeted (AT) and suppression-targeted (ST) muscles after training. As the new muscle synergies were acquired, improvements in the neuromotor control of the trained muscles across the training sessions were observed through the six weeks of the EMG-guided exercise protocol. Therefore, our findings showed the potential of the proposed protocol as a neurorehabilitation strategy after stroke that often induces abnormal intermuscular coordination.

### Development of new intermuscular coordination pattern and its potential effect on motor function

The alterations in the composition or activation profile of muscle synergies have been recently investigated using conditioning or physical training in healthy individuals. For example, Torricelli et al. (2020) examined an EMG-guided conditioning protocol that induced a time shift of activation of muscle synergies in the lower extremity during a cycling task. A study on dynamic arm reaching under a viscous force field applied [50] showed that healthy participants could adapt to the dynamic perturbation by modulating their existing muscle synergies and developing a new pattern of intermuscular coordination. In a recent study by Ghassemi et al. [32] neurologically intact participants demonstrated improved motor control of the hand after receiving EMG-guided training on controlling the activation profile of the existing motor modules. These studies support our findings regarding the feasibility of modulation of muscle synergies through conditioning or training to meet the task demand. Our study further demonstrated the possibility of developing new UE intermuscular coordination patterns through a short-term motor exercise to improve motor output in adulthood.

A few recent studies have further investigated the effects of rehabilitation training on the attributes of muscle synergies in stroke survivors. Pierella et al. [51] showed clinical improvement of stroke-affected UE in the subacute stage by utilizing an assistive exoskeleton in a rehabilitation protocol. Their findings included the clinical improvement, positively correlated with the number of muscle synergies in the trained UE and the similarity of muscle synergy to healthy individuals. Similarly, two studies on stroke survivors with mild-to-moderate impairment demonstrated the increased number of muscle synergies and the similarity of muscle synergy to the control after the rehabilitation training, respectively [52, 53]. These changes in muscle synergies were associated with the improved movement quality of arm reaching. However, the contribution of the increased number or similarity of muscle synergies to improving motor outcomes after stroke is still controversial [54, 55]. Moreover, since the muscle synergy in these studies was used to assess the effect of a given training protocol, not as a target of the training, whether direct targeting of abnormal muscle synergies can improve impaired motor function more effectively remains to be further investigated in stroke.

Considering that muscle synergies can underlie a neural strategy for motor control, expanding the repertoire of the readily available muscle synergies may benefit the motor function of a limb. Based on the findings from the assessment under the “Habitual” condition, there were no noticeable changes in the attributes (i.e., the number, composition, and activation profile) of the habitual muscle synergies throughout the six weeks of the training. However, when the participants intentionally utilize the newly acquired motor strategies (“As-Trained” condition), they could express new intermuscular coordination patterns which were not observed in the habitual muscle synergy set. Interestingly, this expansion of the synergy repertoire did not affect the number of muscle synergies required to perform the same task in either the “Habitual” or “As-Trained” condition. Instead, the expansion was established by the re-structuring of the habitual muscle synergies. The expansion of muscle synergy repertoire can be interpreted as a combination of fractionation (dissociation of BI and BRD in E Flex synergy (Fig. 5)), merging (annexation of BRD to S Add/Flex synergy (Fig. 5)), and conservation of the rest of the synergy compositions. While the muscle coordination complexity remained unchanged after the exercise (i.e., the conserved number of muscle synergies underlying the EMG signals) per assessment task condition, the total number of readily available intermuscular coordination patterns increased by developing new ones. However, to successfully complete the task, not all the available patterns were utilized, but only selectively chosen patterns were used. This phenomenon potentially indicates the increased number of neural strategies for motor control to achieve a given motor task. Thus, we reason that this training effect may benefit motor function after neurological disorders such as stroke by introducing a new intermuscular coordination pattern that can be strategically utilized to compensate for the impaired muscle synergy.

The proposed EMG-guided exercise resulted in changes in not only the composition of muscle synergies but also how the synergies were activated in time (activation profiles of synergies; Fig. 7). Since motor cortical connections [56] and the corresponding descending motor pathway [57] may regulate the muscle synergy activation, the modulation in cortical functional connectivity and regulation of descending pathways due to the exercise may underlie the reformation of the synergy activation pattern. After the stroke, the recovery of impaired motor function is associated with functional brain reorganization [58] and remapping of descending motor pathways [57]. Therefore, the potential effectiveness of the proposed protocol in stroke rehabilitation can be inferred from the following: expanding the repertoire of readily available muscle synergies by developing new ones and modulating the activation profile of the expanded synergy set.

One may raise the question of the effectiveness of the proposed method in neurorehabilitation since it did not affect the habitual muscle synergies. The conservation of habitual synergy observed in this study was potentially due to the biomechanical properties of the targeted muscle pair, BRD and BI. These two elbow flexor muscles are generally coactivated to produce elbow flexion under the isometric condition. However, each also has its own role in shoulder flexion (BI) and forearm supination (BI) or pronation (BRD). This multi-functional nature of muscle activation implies that a neurologically intact human body may already have the ability to learn how to dissociate the BRD-BI activation coupling under certain biomechanical conditions. However, the muscle synergies reflecting the dissociation of BRD-BI could not be considered “habitual” synergies as our results from the participant prescreening showed that 10 out of 12 individuals could not show the dissociation of BRD-BI activation within the elbow flexor synergy even when a verbal tip for facilitating isolated activation of BRD and BI was given. As mentioned in the Methods, only these 10 people were recruited for this study. Therefore, it could be possible for the participants to express a new muscle synergy by learning a new motor strategy that facilitates the isolated activation of a targeted muscle, without affecting their habitual elbow flexor synergy.

In the case of the stroke-induced, abnormally co-activated muscle pair, however, the current protocol may affect impaired (habitual) muscle synergy since a stroke can disrupt the independent controllability of each muscle. Even if the current protocol cannot affect the habitual muscle synergy in the impaired limb after stroke, we expect that the protocol can benefit motor function after stroke by introducing a new intermuscular coordination pattern that can be strategically utilized to compensate for the impaired muscle synergy. In this case, a follow-up training protocol may need to be designed to further adapt the newly developed intermuscular coordination patterns to a variety of functional motor tasks.

### The potential underlying mechanism of developing new intermuscular coordination pattern

The mean frequency shift observed in the EMGs of the dominant muscles in S Add/Flex recorded during the training (Fig. 9) may underlie the potential mechanism of the modulation in muscle synergy. Muscle fatigue is often characterized as a decrease in the center of the EMG power spectrum to lower frequencies [59–61]. Also, the increase in the center frequency of EMG is known to indicate muscle restitution after fatigue [62–64]. From the perspective of muscle synergy, a relatively recent study showed that fatigue of a muscle can influence the attributes of muscle synergy by changing the variance of activation of non-fatigued muscles [35]. Similarly, the findings from a study by Ortega-Auriol et al. [36] demonstrated that muscle fatigue affected the activation profile of the muscle synergies. These fatigue-induced influences may be centrally mediated through adjustments of motor commands [65, 66]. Therefore, our finding, the increased baseline-period EMG frequency of major muscles in S Add/Flex, can be interpreted as a decrease in the fatigue level of the existing muscle in S Add/Flex due to the sharing of the workload with the newly joined muscle, BRD, mediated by CNS [65, 66]. According to the anecdotal report from some participants, they experienced less fatigue in their shoulder region as they acquired new patterns of intermuscular coordination, which supports our interpretation of the results of the frequency analysis.

Based on the results of task performance, the learning curve of “BRD activation-BI suppression” was different from that of the “BI activation-BRD suppression” in the tested experimental design. A previous UE study demonstrated a significantly increased contribution of BRD within BRD-BI coordination during elbow flexion when the hand was pronated [67]. On the other hand, Kleiber et al. [67] also showed that there were no significant changes in the contribution of BI in BRD-BI coordination in any forearm position, which may explain why it took longer for the participants to fully establish the motor strategy for “BI activation-BRD suppression” under the isometric condition. Unlike the BRD-AT trials, the task performance during BI-AT trials was not saturated early in the training period (Figs. 2B and 3B).

### Study limitations and future directions

Although the detailed underlying mechanism of the expansion of the synergy repertoire needs to be further investigated, this study showed that the proposed EMG-guided protocol could induce systematic changes in the composition of a targeted muscle synergy. To investigate this phenomenon more holistically, cortical-, cortico-muscular-, and intermuscular-connectivity associated with the modulation of muscle synergy can be computed using brain imaging and brain stimulation techniques in future studies.

The direct impact of the repertoire expansion of the intermuscular coordination pattern on the activities of daily living (ADLs) remains unexplored. However, to a certain extent, the findings from the present study showed the improvement in the motor control of the trained UE muscles during the training, as well as the transfer of learning of the motor strategies from what was acquired from the training to performing new, untrained tasks during assessment sessions. Although both training and assessment were performed under the same isometric condition, the two tasks were completely different. Therefore, the proposed protocol may benefit UE-related ADLs, which will be examined in our follow-up study.

Another limitation of the current study is that the protocol did not include any retention session after six weeks of the training period since the prime objective of the study was to test the feasibility of developing a new intermuscular coordination pattern. The absence of a control group is another limitation of this study. Our previous study demonstrated that the muscle synergy naturally expressed during an isometric force generation task (the same task performed in the assessment of this study) was conserved across different biomechanical task conditions, experimental protocols, and participants in health [68]. In addition, our preliminary data collected to develop the details of the experimental protocol of the current study showed that the habitual muscle synergy composition in young healthy adults was conserved even after six weeks of the proposed isometric exercise. Based on these findings, including a separate control group in this study seemed unnecessary. However, including a control group in the study could enhance the objectivity of the findings, providing a more objective demonstration of the protocol’s effectiveness. Therefore, additional retention sessions and a control group will be implemented as a part of the protocol in our future study.

When targeting individuals with impaired motor coordination, the current training paradigm may need to be further optimized to minimize its potential risk of disrupting the unaffected coordination of muscles. For stroke, in particular, the characteristics of impaired intermuscular coordination can vary depending on the severity of impairment and time after stroke onset [44]. Therefore, targeting only a pair of muscles may lead to developing a new intermuscular coordination pattern with the undesired coupling of untrained muscles, potentially negatively influencing motor function. One possible way to handle this issue is to carefully identify the abnormality in the muscle synergies (compared to ones in age-matched, healthy individuals or ones in the less affected limb) and utilize activations of a group of muscles as control signals of the cursor for a target match. Moreover, the activation magnitude of the antagonistic muscle groups can be mapped to the cursor movement opposite to the target direction, which may minimize the risk of developing abnormal coactivation of muscles.

The consistency of the sensor location and the baseline posture of participants during EMG-guided tasks is critical, especially for a longitudinal training protocol. Synergy identification relies on electrode placement, in general [69]. In particular, for a longitudinal study like our present work, the inconsistency of EMG electrode placement can cause increased variability in the attributes of the muscle synergy extracted across different sessions. Compression shirts customized to each participant were utilized throughout the study to minimize the potential inconsistency of electrode placement. The locations of the EMG sensors for each participant were identified through the muscle palpation done prior to the study and marked as sensor-size holes on the shirt. Moreover, since the consistency of the posture of the participant during the task can influence the objective investigation of changes in muscle synergy, the angles of each arm joint were strictly measured before and in the middle of the experiment. The upper body movement was constrained with a harness as well.

In addition to the electrode placement, the selection and the number of recorded muscles can affect the muscle synergy analysis [69, 70]. The insufficient number of the selected muscle causes over-estimation of variance accounted for in the data, resulting identification of incomplete muscle synergies [71]. Choosing a larger subset of the recorded muscles can improve the overestimation of explained variance. However, the selection can also increase the possibility of potential EMG contamination by nearby muscle activity (crosstalk). Therefore, selecting an appropriate subset of muscles directly related to the designed motor task is critical. Most previous studies on UE tasks using muscle synergy analysis included between eight to 19 muscles [70]. In our study, we included eight major muscles that were selected in the previous studies that characterized UE muscle synergies during isometric reaching [43, 48, 72] and four additional typical back muscles to examine the contribution of the scapula retractor during the static UE task.

## Conclusions

In summary, this study shows that our isometric EMG-guided protocol can increase the repertoire of readily available muscle synergies within a relatively short period and improve motor control of the trained UE muscles in healthy adults across training sessions. The findings suggest the potential of expanding muscle synergy repertoire as a tool for facilitating motor learning in adulthood, which can be further applied to developing a rehabilitation protocol aiming at a motor deficit induced by neurological disorders. By targeting aberrant intermuscular coordination and developing new ones through EMG-guided exercise, we anticipate improving neurologically impaired patients’ motor performance in activities of daily living.

## Data Availability

The data used in this study may be made available by the corresponding author upon a reasonable request.

## Abbreviations

UE: upper extremity
KULSIS: KAIST Upper Limb Synergy Investigation System
AT: activation-targeted
ST: suppression-targeted
SSR: signal-to-signal ratio
